# Hepatitis C Virus Pathogen Associated Molecular Pattern (PAMP) Triggers Production of Lambda-Interferons by Human Plasmacytoid Dendritic Cells

**DOI:** 10.1371/journal.ppat.1003316

**Published:** 2013-04-18

**Authors:** Amy E. L. Stone, Silvia Giugliano, Gretja Schnell, Linling Cheng, Katelyn F. Leahy, Lucy Golden-Mason, Michael Gale, Hugo R. Rosen

**Affiliations:** 1 Integrated Department in Immunology: University of Colorado Denver and National Jewish Health, Denver, Colorado, United States of America; 2 Division of Gastroenterology & Hepatology, Hepatitis C Center, Department of Medicine, University of Colorado Denver, Aurora, Colorado, United States of America; 3 Department of Immunology, University of Washington, School of Medicine, Seattle, Washington, United States of America; 4 Denver Veteran's Affairs Medical Center, Denver, Colorado, United States of America; McMaster University, Canada

## Abstract

Plasmacytoid Dendritic Cells (pDCs) represent a key immune cell in the defense against viruses. Through pattern recognition receptors (PRRs), these cells detect viral pathogen associated molecular patterns (PAMPs) and initiate an Interferon (IFN) response. pDCs produce the antiviral IFNs including the well-studied Type I and the more recently described Type III. Recent genome wide association studies (GWAS) have implicated Type III IFNs in HCV clearance. We examined the IFN response induced in a pDC cell line and *ex vivo* human pDCs by a region of the HCV genome referred to as the HCV PAMP. This RNA has been shown previously to be immunogenic in hepatocytes, whereas the conserved X-region RNA is not. We show that in response to the HCV PAMP, pDC-GEN2.2 cells upregulate and secrete Type III (in addition to Type I) IFNs and upregulate PRR genes and proteins. We also demonstrate that the recognition of this RNA is dependent on RIG-I-like Receptors (RLRs) and Toll-like Receptors (TLRs), challenging the dogma that RLRs are dispensable in pDCs. The IFNs produced by these cells in response to the HCV PAMP also control HCV replication *in vitro*. These data are recapitulated in *ex vivo* pDCs isolated from healthy donors. Together, our data shows that pDCs respond robustly to HCV RNA to make Type III Interferons that control viral replication. This may represent a novel therapeutic strategy for the treatment of HCV.

## Author Summary

Hepatitis C Virus (HCV) is the most common bloodborne pathogen for which no vaccine is available. Infection with the virus often leads to persistent (or chronic) infection. Patients with chronic HCV infection can develop progressive liver disease and liver failure, leading to the need for a transplant. It is not fully understood why some people clear the virus and others develop persistent infection. Understanding differences in how patients respond to the virus in the early phases of infection may lead to better treatment of HCV. Here, we use a highly conserved region of the HCV genome to examine innate immunological responses to HCV. We found that plasmacytoid dendritic cells, innate cells keyed to respond with anti-viral interferon proteins, recognize the virus. Additionally, we show that pDCs use RIG-I in the recognition of this virus, which was previously thought to be dispensable in pDCs. The proteins secreted by these cells can control viral replication in a cell-based laboratory system. In cells isolated from healthy donors, we found that fresh human cells can respond in the same manner to the virus as the laboratory strain of cells, and there was a correlation with genetic differences. Our study offers novel insight to how the body recognizes HCV during early infection and host-virus interactions that mediate viral control of this common infection.

## Introduction

Pathogens are sensed by host pattern recognition receptors (PRR) that recognize molecular motifs. Two major receptor systems sense the presence of viral infection to mount an immune response: toll-like receptors (TLRs) 3, 7, 8, and 9 are major PRRs that respond to different types of viral nucleic acids, and more recently, retinoic acid inducible gene-I (RIG-I)-like receptors (RLRs), helicases including RIG-I and MDA-5 (melanoma differentiation-associated gene 5), have been identified as cytosolic receptors for intracellular dsRNA sensing [1], [2]. The relative contributions of TLRs and RLRs as viral sensors vary according to viruses and across different cell types [1]. By specializing in the production of Type I Interferons (IFNs), i.e. IFN-α and IFN-β, plasmacytoid DCs (pDCs) play crucial roles as mediators of antiviral responses [3], [4]. RIG-I signaling has been described as largely dispensable for pDC secretion of IFN-α following infection with RNA viruses [1], whereas the TLR system is critical for the RNA virus-mediated IFN response in pDC [5].

Affecting an estimated 200 million people globally, hepatitis C virus (HCV) is the world's most common blood-borne viral infection for which there is no vaccine [6]. The majority of individuals exposed to this RNA virus will develop viral persistence; however, there are significant differences in how patients respond to HCV infection and its treatment [7]. HCV infection has been associated with depletion and functional suppression of pDCs [8], [9]. Some studies have shown that pDCs from the blood of patients with chronic HCV are infected [10], whereas others have failed to demonstrate viral infection within the pDCs. Moreover, some viruses can activate pDCs to produce Type I IFN without the need for active replication [4], [11]. Direct contact with HCV-infected hepatocytes induces Type I IFN via TLR7 signaling within pDCs [12]. Furthermore, Chisari's group has demonstrated that HCV RNA is transferred to the pDCS from hepatocytes via a non-endocytic mechanism [13].

Although their receptor subunits do not display any detectable homology, Type III IFNs are functionally similar to Type I IFNs, signaling through JAK-STAT intracellular pathways and upregulating the transcription of IFN-stimulated genes (ISGs) required to control viral infection [7]. Recently, considerable data have linked genetic variation within or near the Type III IFN-λ3 (IL-28B) gene with HCV recovery [14], [15]. In this study, for the first time, we define the consequences of human pDC sensing of the HCV genome 3′ non-translated poly-U/UC tract, previously shown to function as the HCV pathogen-associated molecular pattern (PAMP) [16] substrate of cytosolic RIG-I. Extensive analysis and characterization of the HCV genomic RNA has identified that the pU/UC tract in the 3′ UTR had the greatest capacity to stimulate IFN-β production in hepatocytes [16]. We hypothesized and now show that the HCV PAMP triggers robust and varied IFN responses from the human pDC cell line GEN2.2 and the secreted Type III IFNs inhibit viral replication. We demonstrate that neutralization of Type III IFNs (IFN- λ) attenuates the anti-HCV effects of pDC-GEN2.2-derived supernatants. Addition of pDC-derived supernatants activates the JAK/STAT pathway in the Huh7.5.1 hepatoma cell line. Furthermore, we demonstrate that laboratory-determined concentrations of Type III IFNs inhibit viral replication. Finally, we recapitulate the induction of Type III Interferons following recognition of the pU/UC RNA in freshly-isolated *ex vivo* human pDCs and show their capacity to inhibit replication.

## Results

### GEN2.2 pDC cell line resembles circulating blood pDCs

**Figure 1:**
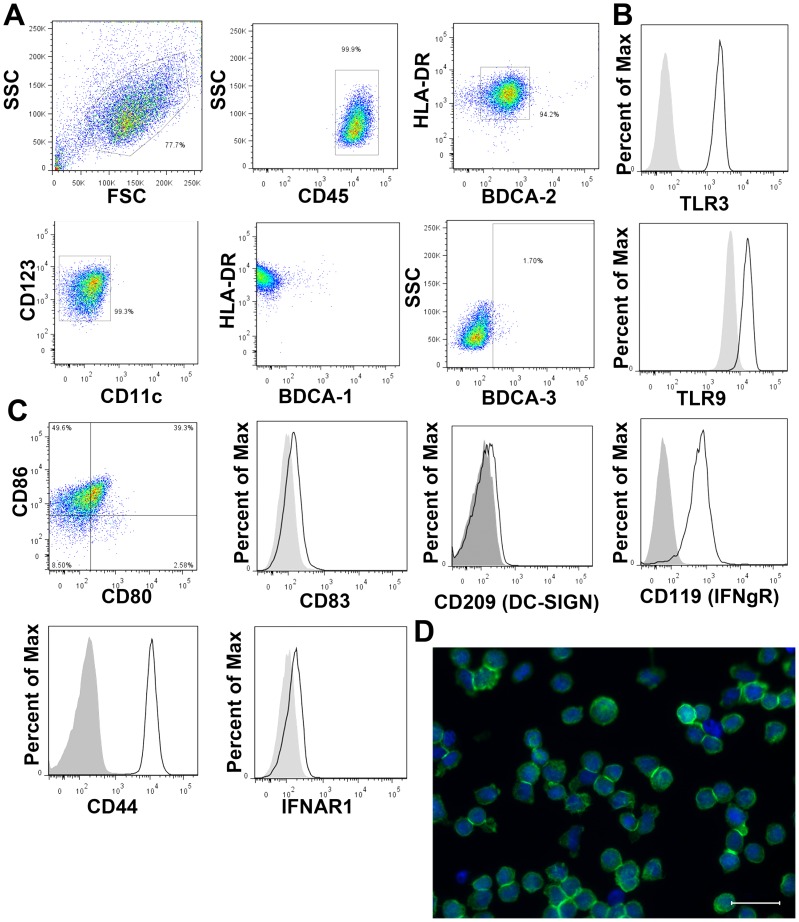
pDC cell line resembles *ex vivo* pDCs. A) Classic pDC surface phenotype. GEN2.2 cell line expresses the classic markers of *ex vivo* human pDCs: CD45+ HLA-DR+ BDCA-2+ CD123+ CD11c− BDCA-1− BDCA-3− (representative flow plots from 3 independent experiments). B) GEN2.2 cell line expresses intracellular TLR3 and TLR9. C) GEN2.2 cell line express costimulatory markers CD86 and CD44, CD119 (IFNγR) and express low levels of CD80, CD83, CD209 (DC-SIGN) and IFNαR1. D) GEN2.2 cellular morphology; Green – β-actin, Blue - Nuclei. Scale bar is 30 µm.

Due to the extremely low frequency of pDCs circulating in normal healthy adults [8], we used the GEN2.2 pDC cell line (pDC-GEN2.2) [17] that phenotypically expresses classic markers of pDCs, as well as various other surface markers relevant to co-stimulation (**Figure 1**). Because our focus was to understand HCV pathobiology, we cultured the pDC-GEN2.2 using the human hepatoma cell line Huh7.5.1 (known to support HCV replication) as the feeder cells. For all of our single-cell experiments using the pDC-GEN2.2 cell line, we used the non-adherent fraction of the cultures. Using only the non-adherent fraction from these cultures, we determined that the cells were over 95% BDCA-2+ and CD45+ thus indicating that the feeder line did not contaminate the cell fraction used for experiments. We felt that using a human hepatic cell line as feeders would make our system more relevant to the study of HCV. The cells were positive for Blood Dendritic Cell Antigen (BDCA)-2 (Cluster of Differentiation [CD] 303), HLA-DR and CD123, markers of pDCs, but were negative for CD11c, BDCA-1 and BDCA-3, typical markers of myeloid DCs. Of the non-adherent cells, over 95% were BDCA-2, HLA-DR double positive. While there is the possibility of contamination from the adherent Huh7.5.1 feeder cells, we feel that this contamination would be minor and not affect the overall results of our studies. Additional characterization of the non-adherent cells showed that these cells expressed CD86, CD44 and CD119 (IFNγR) with low levels of CD80, CD83, CD209 (DC-SIGN) and IFNαR1. TLR2 and TLR7 mRNA was consistently detected, and TLR3 and TLR9 protein levels were demonstrable by flow cytometry. The cells showed a round, even morphology as expected from suspended dendritic cells (**Figure 1D**). The absence of other morphologies within our microscopic samples provides evidence that the non-adherent fraction of our cultures consist primarily of the pDC-GEN2.2 cells. Additionally, we genotyped the cell line for an IL-28B SNP (IFN- λ3; rs12979860) that is associated with HCV clearance [14], [15] and a RIG-I SNP (rs10813831) that has been associated with differential trends in IFNβ1 production [18]. In the case of the IL-28B SNP, the genotype associated with HCV clearance is the CC homozygous allele [14], [15] and was identified as the genotype present in the pDC-GEN2.2 cell line. The RIG-I SNP is an amino acid change (genotype G to A; Arg to Cys) in the protein sequence of RIG-I [18]. Patients with the rare homozygous AA genotype demonstrated a trend of higher RIG-I and IFNβ1 expression levels [18]. The pDC-GEN2.2 cell line was determined to have the AA genotype for this RIG-I SNP. Given the characterization, we concluded that these cells were a reasonable proxy of pDCs for our *in vitro* experiments.

### Robust Type I and III Interferon production following TLR ligation in pDCs

**Figure 2:**
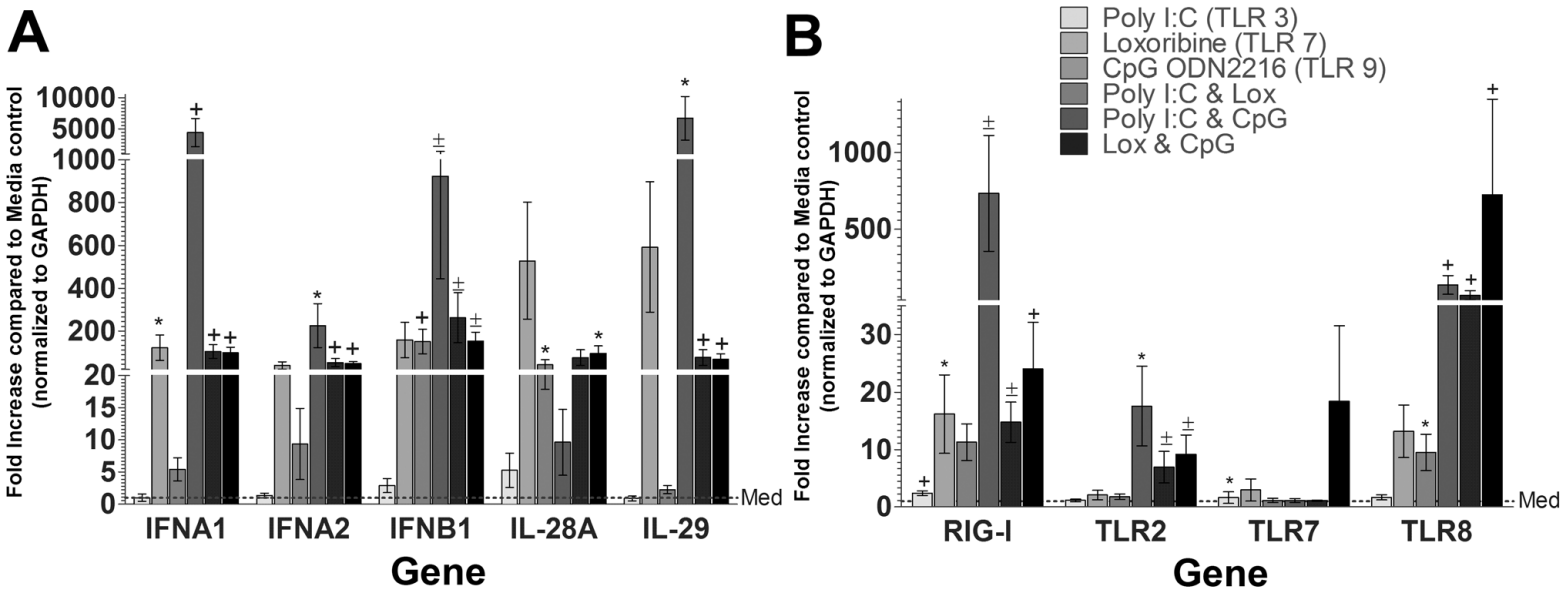
Robust Type I and III Interferon production following TLR ligation in pDC-GEN2.2 cells. Fold increases for each gene at each condition are shown after normalization to reference gene GAPDH and are compared to stimulation of media alone (dashed line). A) Type I and Type III IFN genes are markedly upregulated after TLR stimulation (Type II IFNγ was not expressed; data not shown). The poly I:C and loxoribine combined condition elicited the most pronounced expression, indicating a synergistic effect of TLR3 and TLR7 ligation. B) TLR stimulation induces PRR gene upregulation, most prominently RIG-I and TLR8. p values represent the Wilcoxon signed rank result for each gene and condition as compared to media only condition. * p<0.05+p<0.01±p<0.001 # p≤0.0001. Bars represent the mean and error bars are +/− SEM.

We investigated the profile of IFN transcripts following stimulation with viral TLR ligands *in vitro*. pDCs recognize RNA and DNA viruses through two endosomal sensors, TLR 7 and TLR9, respectively, that induce Type I IFN secretion through the Myeloid differentiation primary response gene (MyD88)-Interferon regulatory factor 7 (IRF7) signaling pathway [4]. By examining the mRNA levels of IFN genes after 6 hours of co-culture with poly I:C (TLR3), Loxiribine (TLR7/8), and Oligodeoxynucleotide (ODN) 2216 (Type A CpG molecule, TLR9 ligand), we characterized ability of the pDC-GEN2.2 cell line to respond to a variety of viral stimulants. As shown in **Figure 2**, there was significant induction of IFNα1, IFNα2, IFNβ1, IL-28A (IFNλ2), and IL-29 (IFNλ1). Not surprisingly, combined TLR3 and TLR7/8 ligation induced the most robust induction of interferon genes given that transcripts for TLR7 were consistently present. Additionally, we found up-regulation of RIG-I, TLR2 and TLR8 mRNA within the pDC-GEN2.2s. Next, we wanted to determine whether a feedback loop of Type I IFN contributed to these IFN expression patterns. To understand how these cells respond to IFNα2 stimulation, we co-cultured the cells with pegylated-Interferon-α2 (commonly used in HCV therapy [19] along with the purine analog ribavirin) for 6 hours and then examined the IFN responses by qRT-PCR. Compared to stimulation with viral TLR ligands, Type I and III IFN showed relatively diminished or absent transcriptional effect, indicating that the response seen after stimulation with viral TLR ligands is not solely attributable to an IFNα2 feedback mechanism (**Figure S1**).

### pDCs sense HCV PAMP and produce Type I and Type III IFNs

**Figure 3:**
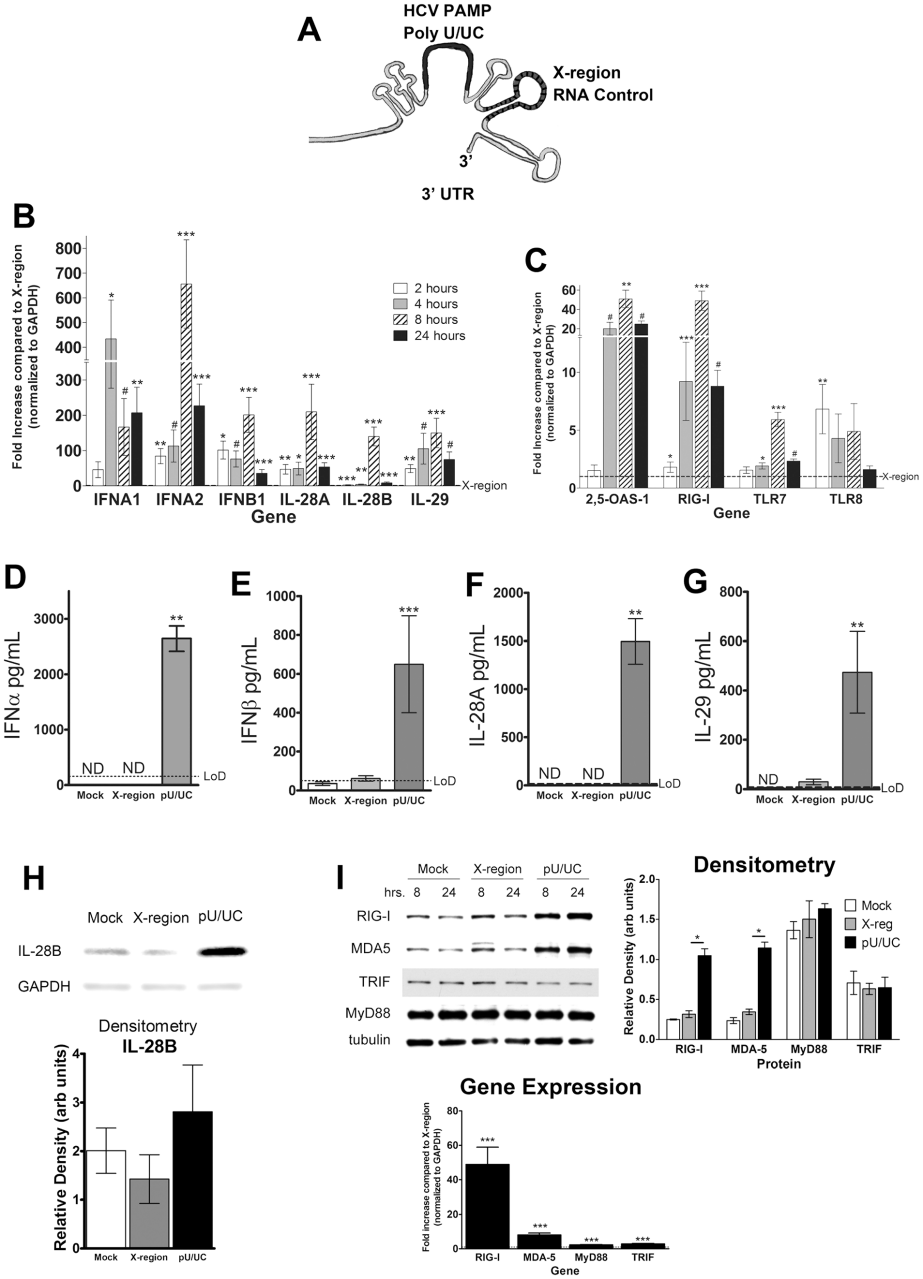
pDC-GEN2.2 cells sense HCV PAMP and produce Type I and Type III IFNs. A) Cartoon of the 3′ end of the HCV genome indicating the location of the poly U/UC (pU/UC, HCV PAMP) and the X-region in the 3′ UTR. Adapted from reference [20]. B) Kinetics of interferon gene upregulation in GEN2.2 cells following transfection with the pU/UC RNA. Fold increases for each gene at each condition are shown after normalization to reference gene GAPDH and are compared to transfection with the X-region RNA (dashed line). Levels of IFN expression at 2 (white bars), 4 (gray bars), 8 (hashed bars) or 24 (black bars) hours are shown for each gene. C) Kinetics of PRR genes and ISGs show upregulation by pU/UC stimulation. Kinetics are shown as indicated in A for the IFN genes. D–G) Secretion of Type I and III IFNs by pDC-GEN2.2 cells following PAMP-stimulation. Increased production of D) IFNα, E) IFNβ, F) IL-28A/IFNλ2 and G) IL-29/IFNλ1 as detected by ELISA from pDC-GEN2.2 cells that have been stimulated with HCV PAMP compared to the X-region RNA. H) Western Blot of pDC-GEN2.2 cell lysates for IL-28B/IFNλ3 after 24 hours of HCV PAMP stimulation. The antibody was specific for IL-28B/IFNλ3 as it recognized low levels of recombinant IL-28B/IFNλ3 (rIL-28B; 10 ng) but failed to recognize recombinant IL-28A/IFNλ2 (rIL-28A; 5 µg). I) pU/UC-stimulation increases PRR signaling proteins in accordance to gene expression data. Western blots of listed PRR proteins after 8 or 24 hours stimulation with HCV PAMP RNA. B–C) Combined data from 5 independent experiments. D–I) Combined data from 3 independent experiments except for the gene expression graphs which are 5 independent experiments. H–I) Representative blots from 3 independent experiments. Densitometry shows relative density of each band after normalization to the reference protein. For gene expression graphs, p values are the Wilcoxon signed rank result for each gene and time point compared to the X-region stimulation from the same gene and time point. For ELISA and Densitometry graphs, p values are the Mann-Whitney result for the pU/UC condition compared to the X-region condition. * p<0.05 ** p<0.01 *** p<0.001 # p≤0.0001 Bars represent the mean and error bars are +/− SEM.

The poly-U/UC (pU/UC) tract of the HCV genome 3′ non-translated region functions as the PAMP substrate of retinoic acid-inducible gene (RIG-I), the cytosolic PRR for HCV [16], [20]. Of the several regions that were tested in the Saito et al. study, this pU/UC region was found to be the most stimulatory region [16]. The nearby X-region, a highly conserved region in the 3′ untranslated region (UTR) of the HCV genome, is not immunogenic and thus was used as a negative control (**Figure 3A**) in these experiments. Transfection of the pU/UC RNA into the pDC-GEN2.2 cells, in the absence of the feeder cells, lead to pronounced transcriptional induction of multiple IFNs (**Figure 3B**), beginning as early as 2 hrs and in general, peaking at 8 hrs, but remaining elevated after 24 hrs of transfection. The X-region RNA did not consistently induce greater IFN production compared to mock transfection (**Figure S2B**). In comparison to the induction of IFN genes by pU/UC RNA, the IFN gene expression induced by the X-region RNA was relatively weak (**Figure S2**). The 2′-5′ oligoadenylate synthetases (OAS) are a family of antiviral proteins that are induced by both virus infection and IFN stimulation and activate latent endoribonuclease (RNase L) [21], yielding RNA cleavage products that initiate innate signaling [22]. HCV pU/UC RNA transfection increased OAS-1 transcription in pDC-GEN2.2 cells more than 40-fold at 8 hrs (**Figure 3C**). However, RNaseL, constitutively expressed in lymphatic tissue [23], was not further induced or upregulated by HCV PAMP transfection (**Figure S3**). Furthermore, transfection with the HCV PAMP upregulated transcriptional expression of PRR signaling genes, in particular RIG-I (**Figure 3C**).

Supernatants from pDC-GEN2.2 cultures that were stimulated with pU/UC RNA, X-region RNA or mock transfection were assayed for levels of IFN proteins by ELISA (**Figure 3 D–G**). At 24 hours post transfection, high levels of all IFN proteins assayed were observed in the pU/UC stimulated condition. The pU/UC IFN protein levels were significantly higher than X-region IFN protein levels for both Type I and III IFNs, in accordance with the gene expression data (**Figure 3B**). The average concentrations of IFNα, IFNβ, IL-28A (IFNλ2) and IL-29 (IFNλ1) were 2600 pg/mL, 650 pg/mL, 1500 pg/mL and 475 pg/mL, respectively. IFNα, IFNβ and IL-29 (IFNλ1) were also detectable at 8 hours post-transfection (data not shown). IL-28B (IFNλ3) could not be assayed by ELISA so we examined IL-28B (IFNλ3) protein levels in cell lysates from the stimulated pDC-GEN2.2 cells by Western blot from three independent experiments. We found that IL-28B (IFNλ3) was detectable in the cell lysates of these cells and that more IL-28B was present in the pU/UC condition than either the mock or X-region condition (**Figure 3H**) although this difference was not statistically significant.

PRR protein levels were also increased after 8 and 24 hours of pU/UC stimulation compared to mock or X-region transfection (**Figure 3I** **, left**). Densitometry revealed a significant increase in protein level for RIG-I and MDA-5 after 24 hours in the pU/UC RNA transfection compared to the X-region RNA transfection (**Figure 3I** **, center**). MyD88 and TRIF protein levels were not increased. The increases in protein are consistent with the gene expression data from 8 hours where each gene showed a significant upregulation from pU/UC stimulation compared to the X-region stimulation with the most robust upregulation in RIG-I and MDA-5 (**Figure 3I** **, right**). Together, these data demonstrate that pDC-GEN2.2 cells not only respond to HCV RNA with IFN gene expression and protein production, but the cells also upregulate PRR genes and proteins, particularly RIG-I which is the known cytosolic receptor for the HCV PAMP.

**Figure 4:**
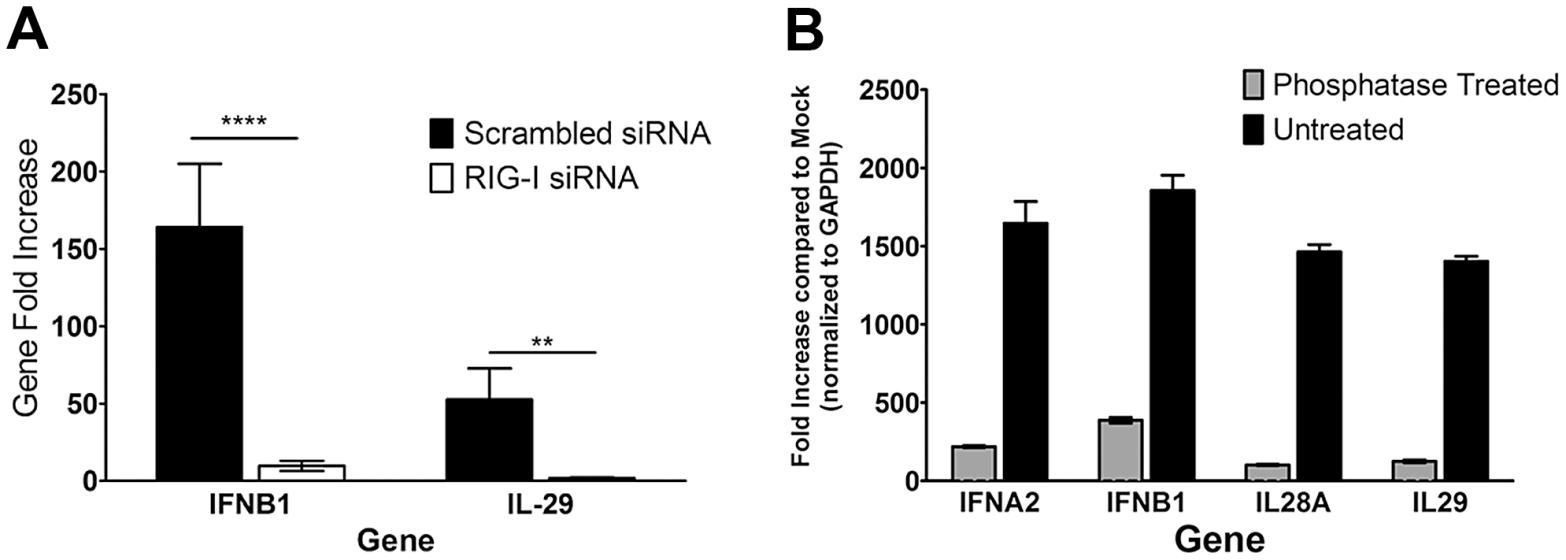
RIG-I contributes to the recognition of the HCV PAMP RNA and is necessary for IFN gene production by GEN2.2. A) Gene expression graphs show that, after normalization to the reference gene GAPDH and Mock transfected condition, when compared to untreated HCV RNA, the phosphatases-treated RNA induced less interferon gene production. Data are representative graph from 3 independent experiments. Bars represent the mean and error bars are +/− SD. B) RIG-I was knocked down in pDC-GEN2.2 cells using siRNA and then stimulated with the pU/UC or X-region RNA. When compared to the Scrambled siRNA condition, the RIG-I knock-down condition (mean knock-down 13% by PCR) produced significantly less IFN mRNA. Combined data from 3 independent experiments. Bars represent the mean and error bars are +/− SEM. p values are the Mann-Whitney result for the scrambled siRNA condition compared to the RIG-I siRNA condition. * p<0.05 ** p<0.01 *** p<0.001 # p≤0.0001.

It has been shown previously that RIG-I requires 5′ triphosphate groups for recognition and signaling [24]. TLR3 and TLR7 have not been shown to have the same requirement for this biochemical feature [25]. We hypothesized that if RIG-I were involved in the recognition and response to the HCV PAMP RNA, removal of the 5′ phosphate groups would abrogate the production of IFN mRNA. We treated the *in vitro* transcribed HCV RNA with Antarctic phosphatases and then transfected 1 µg of the treated RNA into pDC-GEN2.2 cells. After treatment with the phosphatases, much of the IFN mRNA production was lost. This was true of Type I and Type III IFN message (**Figure 4A**). Additionally, siRNA knockdown of RIG-I in the GEN2.2-pDCs resulted in significantly decreased Type I and Type III IFN message after stimulation with the pU/UC RNA compared to the scrambled siRNA condition (**Figure 4B**). These findings suggest that although TLRs have been considered the primary mode of recognition in pDCs, RIG-I is involved in the recognition of the HCV PAMP RNA. While much of the IFN mRNA production was lost, there was still some production of IFN mRNA, suggesting that in addition to RIG-I, other PRRs might be involved in the recognition of this RNA. In particular, TLRs and RLRs may work cooperatively to induce IFN production in pDCs where the division of labor between the PRR systems may be dependent on the stimulating ligand.

### Control of HCV replication in JFH-1/Huh7.5.1 system using conditioned media (CM) from HCV PAMP-stimulated DCs

**Figure 5:**
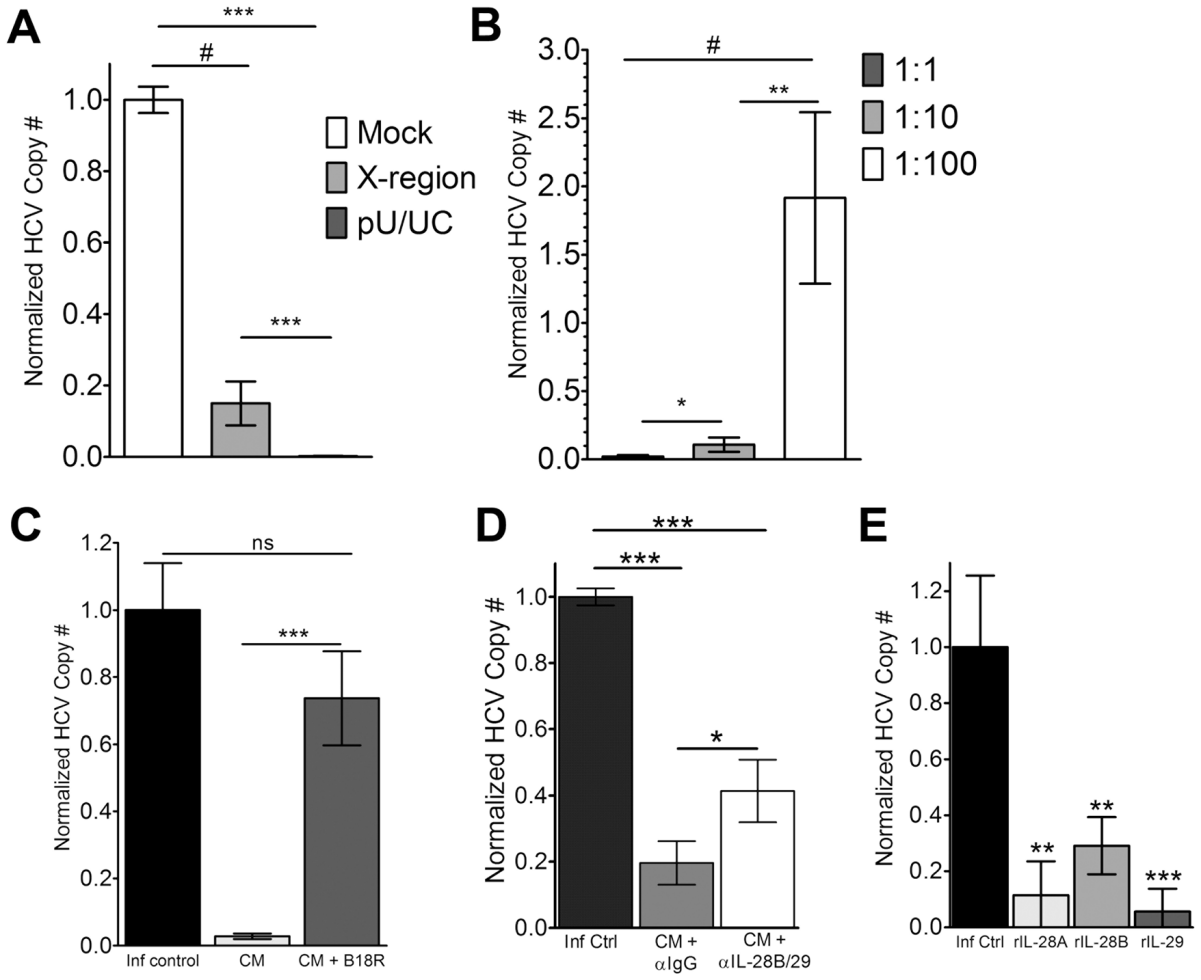
Replicative control of HCV in JFH-1/Huh7.5.1 system with conditioned media (CM) from pU/UC-transfected pDC-GEN2.2 cells. Huh7.5.1 cells were infected for 24 hours prior to the addition of CM (A–D) or rIFNs (E) then 4 days later (5 days post-infection), cells were lysed and examined for HCV Copy number by qRT-PCR (see methods). A) Normalized JFH-1 copy number (see methods and below for calculation) after treatment with CM from Mock (negative; white bars)-, X-region (gray bars)- or pU/UC (dark gray bars)-stimulated pDC-GEN2.2 cells after 8 hours of RNA stimulation. B) Dose-dependent response of viral replication control. CM from the HCV PAMP-stimulated pDC-GEN2.2 cells was added to JFH-1 infected cells at the following dilutions: 1∶1 (Dark gray bars), 1∶10 (gray bars) or 1∶100 (white bars). C) Type I IFN dependence was determined using the Vaccinia protein B18R which blocks Type I IFN responses. D) Blocking IL-28B/IL-29 (IFNλ3/IFNλ1) with a blocking, cross-reactive antibody demonstrates dependence on Type III IFNs for a portion of the viral control. E) Recombinant Type III Interferons in the absence of CM at the same concentrations as found in the CM (IL-28A/IFNλ2: 1500 pg/mL; IL-28B/IFNλ3: 10 pg/mL; IL-29/IFNλ1: 500 pg/mL) were added to JFH-1 infected Huh7.5.1 cells. Normalized HCV Copy Number is shown where the infection control condition HCV copy number is set to 1 (except panel A where the Mock condition is set to 1) and other conditions are expressed as normalized HCV copy number compared to infection control (or compared to Mock in panel A). Normalized HCV Copy Number = (Absolute copy number for condition/absolute copy number for infection control). Combined data from 3 (A, C and E), 5 (B, D) independent experiments p values represent the Mann-Whitney result of the comparison. * p<0.05 ** p<0.01 *** p<0.001 # p≤0.0001. Bars represent the mean and error bars are +/− SEM.

In order to test the ability of the pDC-GEN2.2 cells to control viral replication in hepatocytes, we used the JFH-1/Huh7.5.1 *in vitro* culture system [26]. Cell-free supernatants from pDC-GEN2.2 cells that had been transfected with the pU/UC RNA were added to Huh7.5.1 cells 24 hours after infection with the HCV JFH-1 virus. Four days later, Huh7.5.1 cells were lysed and the viral copy number was assayed by qRT-PCR. We found that CM from pDC-GEN2.2 cells transfected with the pU/UC RNA controlled viral replication better than CM from pDC-GEN2.2 cells transfected with the X-region RNA or mock-transfected (**Figure 5A**). Moreover, this effect was dose-dependent. The viral control seen with the CM was attenuated at the 1∶10 dilution and lost at the 1∶100 dilution (**Figure 5B**). In order to identify which signaling pathways were activated within hepatocytes following co-culture with CM from pDC-GEN2.2 cells, we used a JAK/STAT pathway gene expression array (**Table S1**). Huh7.5.1 cells were infected for 24 hours, and then CM was added at a 1∶1 dilution. Sixteen hours after the addition of the conditioned media, we isolated RNA and examined the gene expression using the JAK/STAT pathway gene expression array. The time-point of 16 hours was selected since the peak for Type I IFNs is 6 hours and the peak for Type III IFNs was 24 hours [27]. The most up-regulated genes were STAT1 and IRF-9 (ISGF3G), a key regulator in the JAK/STAT pathway, and these were confirmed by individual qRT-PCR (**Figure S4**).

Next, we focused on defining the relative contribution of Type III IFNs secreted by the pDC-GEN2.2 cells to viral control. In our model system, at the same time as the addition of the 1∶1 dilution of CM from the pDCs to the infected Huh7.5.1 cell cultures, we added a blocking antibody against Interferon-λ1/λ3 (IL-29/28B) or an isotype control antibody or the Vaccinia virus protein B18R. Using the Vaccinia virus protein B18R, which only blocks Type I IFNs and does not recognize IFNλ [28], viral control induced by the CM was eliminated (**Figure 5C**). When blocking the Type III IFNs with the dual blocking antibody, viral control was impaired. In the presence of CM and the isotype antibody, there was greater than 74% viral control, and this effect was partially lost when the dual blocking antibody was present (**Figure 5D**). This finding suggests a role for Type III Interferons in the control of viral replication. This was further confirmed by addition of recombinant Type III IFNs in the absence of CM at levels comparable to those detected within the supernatants of HCV PAMP-transfected pDC-GEN2.2 cells (**Figure 5E**); all recombinant Type III IFNs alone demonstrated viral control at these experimentally-determined concentrations.

### Co-culture of GEN2.2-pDCs and infected Huh7.5.1 cells leads to viral control and production of Interferons

**Figure 6:**
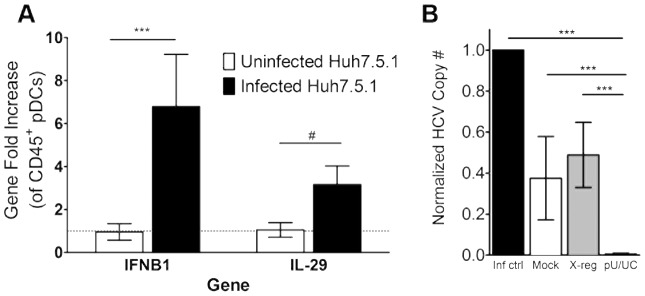
Co-culture of JFH-1-infected Huh7.5.1 cells and pDC-GEN2.2 cells leads to the upregulation of IFN genes and viral control. A) Huh7.5.1 cells were infected for 24 hours with JFH-1 and then resting pDC-GEN2.2 cells were added for 24 hours. mRNA from the CD45+ cells was isolated and examined for IFN gene expression. Gene fold increase is shown for each gene after normalization to the reference gene GAPDH and the uninfected condition. B) Huh7.5.1 cells were infected for 24 hours with JFH-1 and then pDC-GEN2.2 cells that had been mock transfected, transfected with X-region RNA or pU/UC RNA for 8 hours were added for 4 days (5 days total infection). RNA was isolated and examined for JFH-1 copy number. Normalized HCV copy number is shown where the infection control condition HCV copy number is set to 1 and other conditions are expressed as normalized HCV copy number compared to infection control. Normalized HCV Copy Number = (Absolute copy number for condition/absolute copy number for infection control) p values represent the Mann-Whitney result of the comparison. * p<0.05 ** p<0.01 *** p<0.001 # p≤0.0001. Bars represent the mean and error bars are +/− SEM.

In order to address the ability of the pDC-GEN2.2 cells to respond to intact virus and infected Huh7.5.1 cells we co-cultured the pDC-GEN2.2 cells with infected Huh7.5.1. After 24 hours of exposure to the infected Huh7.5.1 cells, the pDC-GEN2.2 cells had significantly upregulated Type I and Type III IFNs compared to co-culture with uninfected Huh7.5.1 cells, represented by IFNB1 and IL-29/IFNλ1 (**Figure 6A**). In contrast to the HCV PAMP stimulation, we did not see upregulation of RIG-I by PCR (data not shown). Additionally, infected Huh7.5.1 cells were co-cultured with pDC-GEN2.2 cells that had been transfected with the pU/UC tract RNA and examined for HCV copy number. While the presence of pDC-GEN2.2 cells alone did lead to a reduction in viral copy number, co-culture with pDC-GEN2.2 cells transfected with the pU/UC tract RNA controlled virus significantly better than pDC-GEN2.2 cells that were transfected with the X-region RNA or mock-transfected (**Figure 6B**).

### Primary circulating pDCs respond in a similar manner to the HCV RNA PAMP

**Figure 7:**
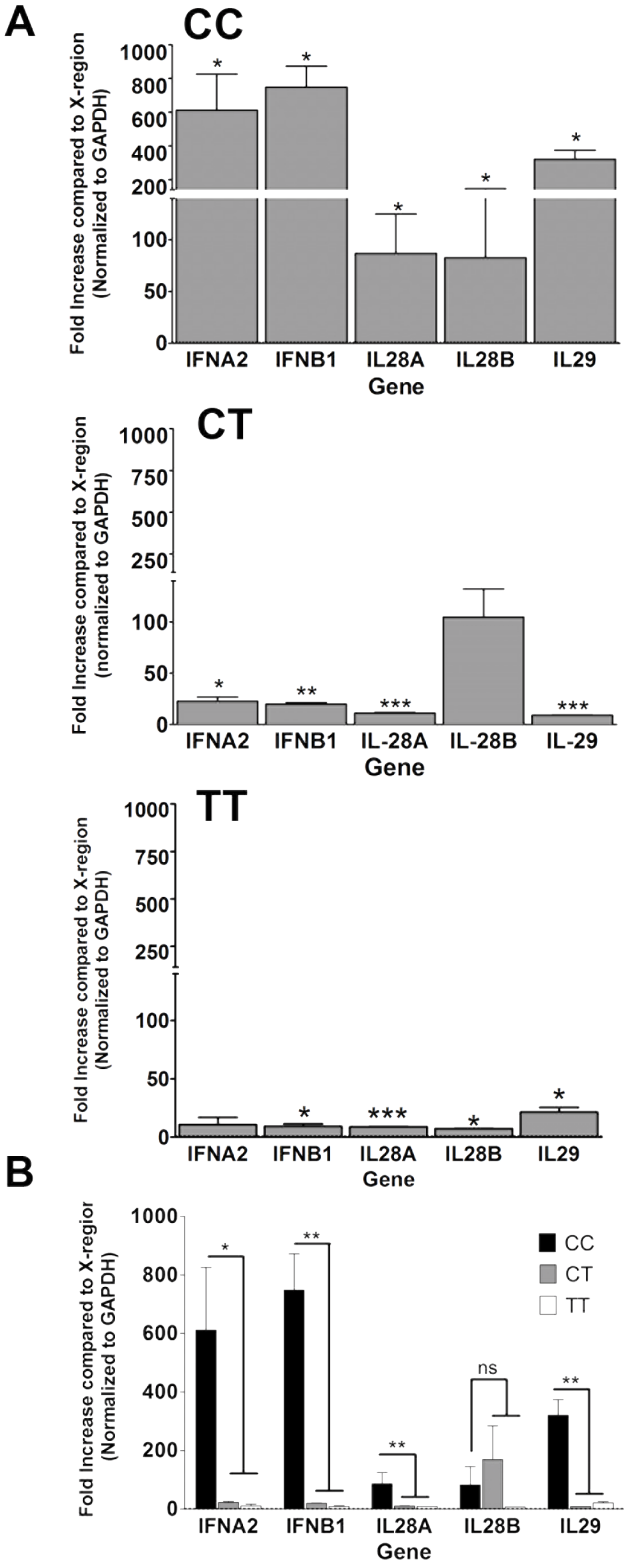
*Ex vivo* pDCs upregulate Type I and III Interferon genes in response to the HCV PAMP. A) Gene expression changes in *ex vivo* pDCs after HCV PAMP RNA stimulation. Top: subjects with the CC IL28B/IFNλ3 genotype (2 subjects IL28A/IFNλ2, IL28B/IFNλ3, IL29/IFNλ1 and IFNβ1; 1 subject IFNα2; RIG-I SNPs GG and GA); middle: CT IL28B/IFNλ3 genotype (1 subject, RIG-I SNP AA); Bottom: TT IL28B/IFNλ3 genotype (1 subject RIG-I SNP GG). Top graph is combined data from 2 independent experiments while middle and bottom graphs are data from a single independent experiment each. B) Same gene expression data as in A graphed together. Compared to the non-CC genotypes, the CC subjects had significantly more IFN mRNA. p values are the Wilcoxon signed rank result for (A) each gene compared to the X-region stimulation (dashed line) from the same gene, and (B) each gene CC genotype compared to each gene non-CC genotype. * p<0.05 ** p<0.01 *** p<0.001 # p≤0.0001. Bars represent the mean and error bars are +/− SEM.

We wanted to confirm the effects seen in the pDC-GEN2.2 cell line in response to the HCV PAMP in *ex vivo* human pDCs. To accomplish that, we isolated pDCs from four normal healthy donors (see Methods). We characterized the pDCs using flow cytometry (**Figure S5**) and found these cells to be 95+% pure with little contamination from natural killer cells, T cells, B cells or monocytes. These cells also expressed low levels of the co-stimulatory markers CD80 and CD86 but highly expressed CD44. After stimulation with the HCV RNA in the exact same way as the pDC-GEN2.2 cell line (1 µg of HCV PAMP RNA, either pU/UC or X-region RNA, was transfected into the cells; RNA was isolated 8 hours post transfection and assayed for expression of IFN genes), the *ex vivo* pDCs significantly upregulated IFNα2 (except the TT subject), IFNβ1, IL-28A (IFNλ2), IL-28B (IFNλ3; except the CT subject) and IL-29 (IFNλ1) compared to the X-region RNA (**Figure 7A**). Due to the importance of the IL-28B (IFNλ3) SNP to HCV clearance, we selected subjects with each of the 3 possible genotypes to test. The subjects with the favorable CC genotype had over 100 fold more robust responses to the pU/UC RNA than the subjects with the CT or TT genotype. Additionally, the CC genotype subjects' gene expression levels were significantly higher than the non-CC genotype subjects' gene expression level in all cases except IL28B/IFNλ3 (IFNα2 p<0.05, IFNβ1 p<0.01, IL28A/IFNλ2 p<0.01, IL28B/IFNλ3 n.s., IL29/IFNλ1 p<0.01; **Figure 7B**). While the TT genotype had the least robust response, the IFN genes were still significantly upregulated compared to the X-region RNA control. One of the reasons for the difference between the cell line Interferon levels and the non-CC *ex vivo* cell Interferon levels may be that the cell line has both of the “favorable” genotypes for IL-28B/IFNλ3 (rs12979860; CC) [14], [15] and RIG-I (rs10813831; AA) [18] while the non-CC subjects have the “unfavorable” TT or CT for the IL-28B/IFNλ3 SNP and AA or GG for the RIG-I SNP. Though the AA RIG-I genotype is considered to be “favorable”, the presence of that favorable allele was not enough to overcome the “unfavorable” IL-28B/IFNλ3 SNP. These data confirm that *ex vivo* pDCs are capable of responding robustly to HCV RNA with Type I and III Interferon production.

The supernatants from these cells were assayed for IFNα and IL-29 (IFNλ1) by ELISA (**Figure S5A** and **S5B**). Each subject's pDCs had more IFN present in the pU/UC-transfected condition compared to the X-region-transfected condition. Conditioned media from the transfected primary *ex vivo* pDCs were used to treat infected Huh7.5.1 cells in the same manner as previously described for the pDC-GEN2.2 CM. The pU/UC-transfected CM from the CC subjects (but not the non-CC subjects) controlled virus better than X-region-transfected CM from those same subjects (**Figure S5C**).

## Discussion

Dendritic cells play a critical role as innate pathogen sensors [29]. Although they constitute only 0.2% to 0.8% of peripheral leukocytes in healthy subjects, pDCs have been found to produce over 95% of Type I IFNs by PBMCs in response to many viruses [29]. HCV represents the most common blood-borne viral infection for which there is no vaccine [6]; acute infection is followed by development of viral persistence in the majority of subjects. In this study, we comprehensively characterized the response to transfected hepatitis C viral PAMP using the pDC cell line GEN2.2. As with any cell line, there were some differences between the cell line and *ex vivo* cells. In keeping with prior reports [30], we did not find freshly isolated human *ex vivo* pDCs to express TLR3 (**Figure S5**); in contrast, the pDC cell line did express TLR3. However, for the most part, the cell line resembled *ex vivo* cells.

The current dogma is that TLR and not RLR signaling predominate within the pDC population [5]. In this study, TLR ligand stimulation of pDCs increased transcription of Type I and III IFNs, as well as PRRs. These data suggest that TLR ligation not only induces IFN, but also primes the cells for recognition of additional viral stimuli. This type of priming has not been thoroughly investigated for viruses although the presence of bacterial products such as LPS have been shown to prime various innate immune cells, primarily neutrophils [31] and macrophages [32]. A feedback mechanism of TLR3 upregulation by Type I IFNs to amplify the response to TLR3 ligands was previously proposed [33]. It may also be the case that viral stimulation primes pDCs for additional recognition and enhanced IFN production. When we stimulated the cells with PEG-IFN-α2, we failed to induce Type III IFNs, data that are consistent with a recent study using primary human hepatocytes [27]. In accordance with prior reports, we found that the cytosolic receptor RIG-I was IFN-inducible [5]. We speculate that the induction of the IFN genes may lead to an antiviral state within *in vivo* pDCs thus making them resistant to HCV infection and prone to rapid clearance of the virus within the pDCs themselves. This may explain why HCV RNA is not consistently found within circulating pDCs from HCV-infected patients.

The pDC cell line produced IFN in response to pU/UC RNA, but not X-region RNA, with most genes peaking in expression at 8 hours, consistent with the known kinetics of IFN gene expression [34]. Given that the pU/UC RNA was generated using an *in vitro* transcription system, one potential concern was that the 5′ triphosphate added by the T7 polymerase might artificially induce IFN responses. However, since the X-region RNA was made in the same way (and thus had the same triphosphate), but failed to induce IFN production (**Figure S2**), we felt confident that the gene and protein expression levels that we observed were due to pU/UC RNA-specific features.

In this study, transfection was used to introduce HCV RNA into the pDC-GEN2.2 cells. Previous work has demonstrated that pDCs can sense HCV and respond with IFNα production in an *in vitro* system using Huh7.5.1 c2 cells and JFH-1 requiring cell-to-cell contact [13]. Chisari's group also showed that receptor-mediated entry of the virus into the pDCs was not required to induce IFN production [13]. This latter study provides rationale for using transfection to mimic the non-receptor mediated transfer of RNA from hepatocytes to pDCs. We found that HCV PAMP transfection of pDCs induced an antiviral effect when co-cultured with infected hepatocytes. In keeping with and extending Takahashi's study, we found that co-culture with infected Huh7.5.1 induced transcriptional upregulation of Type I and III IFNs. There are a number of means by which pDCs could sense *in vivo* infected hepatocytes, including encounter of viral RNA in debris from infected cells killed by immune mechanisms, such as natural killer cells, or from endocytosed virions. Transfection acts as a laboratory tool to imitate viral RNA transfer *in vivo*.

Viral control in our replication model system related to the conditioned media (CM) from pU/UC RNA-transfected pDCs was partially mediated by Type III IFNs. This is in agreement with previous reports that IFNλs can control HCV *in vitro* [35]. We also demonstrated that STAT1 and IRF9 were upregulated by the addition of pU/UC-transfected CM, but not X-region-transfected CM, which implicate a role for IFNs in mediating the viral control within the infected hepatocytes. At the same concentrations as detected in the CM, Type III IFNs were able to control HCV replication. Both IL-28A (IFNλ2) and IL-29 (IFNλ1) at higher concentrations (100 ng/ml) have been shown to significantly reduce HCV replication with the same efficacy and comparable to IFNα [36]. In light of prior work indicating that viral transcription is not required for RIG-I activation [37], our collective data suggest an IFN-mediated mechanism that provides inhibition of HCV replication during the initial stages of infection [35], [38]. Human pDC sense conserved regions of HCV RNA, resulting in the upregulation of the cytosolic RIG-I- helicase, ISGs, and robust production of Type III IFNs that mediate antiviral activity in HCV-infected hepatocytes.

The current observations also help shed light on the puzzling lack of association between IL-28B/IFNλ3 SNPs and hepatic expression of Type III IFNs reported in the literature [7]. Our data using both a cell line and freshly isolated *ex vivo* cells suggest that pDCs sensing HCV RNA produce different levels of IFN, perhaps indicating a functional role of the polymorphisms in the IL28B/IFNλ3 gene locus. Hepatic mRNA (in prior studies) would have been derived predominantly from hepatocytes, whereas the main cellular sources of Type III IFNs are pDCs. Interestingly, pDCs derived from subjects with the favorable CC genotype also demonstrate significantly more robust Type I IFN transcription following HCV PAMP stimulation that may be the result of an amplification feedback loop or modification of transcriptional factors. Clearly, further work is warranted to understand the mechanisms of how Type I and III IFNs might be co-regulated. Induction of RIG-I mediated responses by the use of synthetic agonists may have implications for novel therapeutic approaches for this common infection. Moreover, mechanisms that antagonize RIG-I-mediated stimulation of IFN warrant further investigation in order to understand why the majority of subjects develop viral persistence.

## Materials and Methods

### Ethics statement

Patients were recruited and written informed consent was obtained using COMIRB approved protocol # 08-0364 from the Denver Metro area.

### Cell culture

GEN2.2 cell line was grown on Huh7.5.1 cells in either DMEM with 10% Human Serum+1X Pen/strep+1X NEAA (Non-essential Amino Acids) or RPMI with 10% FBS+1X Gentamycin. No differences in the functionality of the cells were seen based on media condition (Data not shown). Briefly, Huh7.5.1 cells were plated and allowed to grow until 90% confluency then the GEN2.2 cells were added. For experiments, the non-adherent fraction of the culture was used. These cells were over 95% BDCA-2+ and CD45+ thus indicating that the feeder line was not included in these experiments.

### Flow cytometry

#### Surface stain

Cells were washed with FACS wash (PBS with 0.016% sodium azide, 0.6% BSA). Cells were then resuspended in FACS wash containing fluorescently labeled antibodies (BDCA-2, BDCA-3, BDCA-4 and BDCA-1: Miltenyi Biotech. Lineage cocktail, CD123, HLA-DR, CD86, CD80, CD83, CD209, CD45, CD44, CD119, TLR9, CD93w: BD Biosciences. CD11c, and TLR3: eBiosciences. IFNAR1: R&D Systems) and incubated at 4C for 30 minutes. Cells were then washed twice in FACS wash and resuspended in 2% PFA in PBS for 20 min. Cells were acquired within 24 hours on BD FACSCanto II. Data was analyzed using FlowJo software. Intracellular stains: Cells were stained for surface antigens as described above until second wash after antibody incubation. Cells were then resuspended in BD Perm Buffer III (BD 558050) for 30 minutes then pelleted and resuspended with intracellular antibodies in BD Perm Buffer III for 30 minutes. Cells were then pelleted and resuspended in 2% PFA for 20 min. Cells were acquired within 24 hours on BD FACSCanto II. Data was analyzed using FlowJo software.

### Fluorescent microscopy

Cells were fixed in 4% PFA for 30 minutes. Cells were then washed in DPBS and resuspended in blocking buffer (3% BSA, 10% FBS, 0.3% Triton X-100 in DPBS) for 30 minutes at room temperature. Cells were then washed in DPBS and resuspended in Phalloidin conjugated to AlexaFluor 488 (Invitrogen A12379) in Binding Buffer (10% FBS, 0.3% Triton X-100 in DPBS) for 30 minutes in the dark at room temperature. Cells were then washed in DPBS and resuspended in DPBS. Cells were cytospun on the slide by using adding the cell suspension to a cytofunnel and spun at 1000 rpm for 5 min. The slide was allowed to dry at room temperature in the dark. Mounting media containing DAPI (Vector Labs H-1200) was added to the slides and covered with a coverslips and sealed. Slides were viewed on a Leica DM5000B with a Leica DFC350FX camera. The objective lenses were Leica 506507 (10X/0.30 NA), Leica 506506 (20X/0.50 NA) and Leica 506145 (40X/0.75 NA). Images were taken at room temperature (23°C) and acquired using Leica FW400 software. Images were not adjusted or modified after image capture.

### TLR and IFNα stimulation

Cells were plated in a 24 well low-adherence plate (Costar 3473) at a concentration of 1×10^6^ cells in 1 mL per well. TLR ligands, IFNα or media were added: either alone or in combination: Poly I:C [100 µg/mL, Sigma P0913], ODN 2216 CpG [250 µM, Invivogen tlrl-hodna], Loxiribine [1 mM, Invivogen tlrl-lox], PEG-IFNα [100 ng/mL]. The cells were incubated at 37C, 5% CO_2_ for 6 hours at which time RNA was isolated using RNeasy Mini kit (Qiagen, 74106) with the Qiashredder option (Qiagen, 79656). RNA was quantified using a Nanodrop microspectometer. 1 µg of RNA was used to make cDNA using Quantitect RT kit (Qiagen, 205313). qRT-PCR was performed using SYBR Green primers and master mix from Qiagen and run on a StepOnePlus qPCR machine from Applied Biosystems. Data was analyzed by the ΔΔCT method. All primers used in the qRT-PCR assays were purchased from Qiagen.

### HCV PAMP preparation

pU/UC and X-region plasmids were kindly provided by Dr. Michael Gale's lab. The plasmids were amplified using PCR (X-region Forward 5′-TAATACGACTCACTATAGGTGGCTCCATCTTAGCCCTA-3′ X-region Reverse 5′-ACTTGATCTGCAGAGAGGCCAGTATCA-3′ HCV pU/UC Forward 5′-TAATACGACTCACTATAGGCCATCCTGTTTTTTTCCC-3′ HCV pU/UC Reverse 5′-AAAGGAAAGAAAAGGAAAAAAAGAGG-3′) with a high fidelity polymerase (Invitrogen 11304-011). The PCR products were separated with electrophoresis on an (1%) agarose gel. The bands of interest were extracted from the gel (Gel extraction kit, Qiagen 28704) and transcribed *in vitro* (Applied Biosystems AM1354M). The final product was quantified using a Nanodrop microspectometer. For removal of the 5′ Phosphate groups, the HCV PAMP RNA was treated with Antarctic phosphatases (New England Biolabs, M0289) as per manufacturer's instructions. The enzyme was deactivated for 5 min at 65C. This RNA was then used as described for the untreated HCV PAMP (see below).

### HCV PAMP stimulation

Cells were plated in a 24 well low-adherence plate at a concentration of 1×10^6^ cells in 1 mL per well. 1 µg of pU/UC, or X-region RNA or 1 µL water (mock) were transfected (Mirus 2250) for 2, 4, 8 or 24 hours at 37C, 5% CO_2_. 1 µg of RNA was used because 1 µg of HCV PAMP RNA was used by Dr. Gale's group in their original study of the immunogenic capacity of these RNAs [16]. RNA was isolated, cDNA was generated and qRT-PCR was run and analyzed as described for TLR stimulation. Supernatants were also collected at 2, 4, 8 or 24 hours post-transfection.

### siRNA knockdown

Cells were plated in a 24 well low-adherence plate in 500 µL/1×10^6^ cells serum-free Accell Delivery Media (Dharmacon # B-005000). siRNA was then added to a final concentration of 750 nM. Cells were incubated for 48 hours at 37C, 5% CO_2_. Accell Delivery Media+10% Heat-inactivated FBS (500 µL/1×10^6^ cells) was added to the wells. Cells were incubated for an additional 24 hours and then stimulated as described above. DDX58 (RIG-I) siRNA (Smart Pool; E-012511-00) and scrambled siRNA (Non-targeting control; D-001910-10) were obtained from Fisher Scientific.

### ELISAs

ELISA kits for IFNα (PBL Interferon Source, 41105), IFNβ (PBL Interferon Source, 41410), IL-28A/IFNλ2 (RayBio, ELH-IL28A-001) and IL-29/IFNλ1 (eBiosciences, 88-7296) were used as per the manufacturer's instructions. All samples used at either a 1∶1 or 1∶10 dilution and were incubated overnight at 4C.

### Western blots

Cell lysates were prepared by stimulating cells as described above. Cells were harvested on ice and washed twice with cold PBS. Cells were then lysed with a Modified RIPA buffer (Tris-HCl 50 mM, 1% NP-40, 0.25% Na-deoxycholate, NaCl 150 mM, EDTA 1 mM pH 7.4) with 1X protease inhibitors (Roche 11 836 170 001) and 1X phophatase inhibitors (Fisher Scientific, PI-78420). After 30 minutes of agitation at 4C, cell lysates were centrifuged and pellets were discarded. Protein levels were assayed using a BCA assay (Fisher, PI-23221 and PI-23224) as per manufacturer's instructions. Samples were separated using SDS-PAGE on Mini-protean TGX Any kD gels (Bio-rad, 456-9033) and transferred onto a nitrocellulose membrane using a wet transfer system. Membranes were blocked for 1 hour with 5% milk in TBST or PBST, washed, and proteins were analyzed by immunoblotting with standard methods using antibodies specific to RIG-I (Enzo Lifesciences), MDA5 (Cell Signaling Technology), MyD88 (Cell Signaling Technology), IL-28B/IFNλ3 (R&D Systems), RNase L (Thermo Scientific), TRIF (Cell Signaling Technology), tubulin (Sigma), and GAPDH (Abcam). Secondary antibodies conjugated to HRP were obtained from Jackson ImmunoResearch and Cell Signaling Technology, and immunoreactive bands were detected with either the Immuno-Star HRP Substrate kit (Bio-Rad, 170-5040) or the Amersham ECL Prime Western Blotting Detection Reagent (GE Healthcare). Densitometry was performed using ImageJ (NIH), and proteins of interest were normalized to a reference protein (Tubulin or GAPDH).

### RNA gels

Whole RNA was isolated from HCV PAMP stimulated cells as described above. RNA was run on a 1% agarose formaldehyde gel with Ethidium bromide. Gel was visualized via UV.

### Viral control assays

Huh7.5.1 cells (kindly provided by Frank Chisari, Scripps) were plated in a 24 well plate at a concentration of .125×10^6^ cells per well in 1 mL media overnight. The cells were then infected with JFH-1 at an MOI (multiplicity of infection) of 0.01. Twenty-four hours following infection, the supernatants generated from the HCV PAMP transfection (either Mock (negative) transfection, X-region or pU/UC) were added at various dilutions. Five days post-infection the supernatants were aspirated and RNA was isolated as previously described. HCV copy number qRT-PCR was performed using JFH-1 primers (JFH-1 Forward 5′-CGACACTCCGCCATGAATCACT-3′ and JFH-1 Reverse 5′-CACTCGCAAGCGCCCTATCA-3′) and TaqMan TAMRA probe (5′-6FAMAGGCCTTTCGCAACCCAACGCTACTTAMRA-3′). The copy number was determined using a standard curve. Using the infection control condition absolute HCV copy number all other conditions' copy numbers were normalized for that particular experiment. The following formula was used to determine Normalized HCV Copy Number: Normalized HCV Copy Number = (Absolute copy number for condition/absolute copy number for infection control). A blocking antibody against IL-28B/IL-29 (IFNλ3/IFNλ1) or an isotype control antibody were obtained from R&D Systems (10 µg/mL, MAB15981, MAB003) or the recombinant Vaccinia protein B18R from eBioscience (0.1 µg/mL; 14-8185-62) and added to the supernatants at 24 hours post-infection and the experiment was performed as described. Recombinant Interferon proteins (IL-28A/IFNλ2, PeproTech, 300-02K: 1500 pg/mL; IL-29/IFNλ1 PeproTech, 300-02L: 500 pg/mL; IL-28B/IFNλ3, R&D Systems, 5259-IL-025: 10 pg/mL) were added to the infected Huh7.5.1 cultures 24 hours post-infection and the experiment was performed as described above. For the JAK/STAT pathway gene expression arrays (Applied Biosystems, 4414156) and validation Huh7.5.1 cells were plated, infected and treated with pDC supernatants as described above. Sixteen hours after the addition of the supernatants, the cells were harvested for RNA as previously described. cDNA was made and the gene expression array plates were run as per manufacturer's instructions or individual genes were examined by qRT-PCR as described above. For co-culture experiments, Huh7.5.1 cells were plated overnight and infected as described above. The non-transfected pDC-GEN2.2 cells were added 24 hours post-infection. Cells were co-cultured for 24 hours and then the non-adherent fraction was collected for qRT-PCR analysis. This fraction was examined for BDCA-2 expression and 98+% were BDCA-2+ indicating little to no contamination of the Huh7.5.1 cells. For the co-culture experiments with the transfected pDC-GEN2.2, Huh7.5.1 cells were plated and infected as described above. Twenty-four hours post-infection, pDC-GEN2.2 cells were transfected with HCV PAMP RNA as described previously in the methods (8 hour transfection) and added to infected Huh7.5.1 cells. As described above for pure Huh7.5.1 cultures, the cultures were examined five days post-infection for HCV copy number by qRT-PCR.

### pDC isolation from whole blood

Patients were recruited and consented using COMIRB approved protocol # 08-0364 from the Denver Metro area. PBMCs from normal, healthy patients were screened for pDC percentage of >0.25% of total PBMCs using Lineage^negative^, HLA-DR^+^, BDCA-2^+^ and BDCA-4^+^. Patient cells were also genotyped for IL28B and RIG-I SNPs (rs12979860 and rs10813831, respectively). Patients were leukopheresed and pDCs were immediately isolated using Miltenyi's Diamond Plasmacytoid Dendritic Cell Isolation Kit II (Miltenyi Biotech, 130-097-240) and characterized using flow cytometry and fluorescent microscopy as described for the pDC cell line. Cells were then stimulated in the exact same way as the pDC-GEN2.2 cell line with the HCV PAMP RNA (1 µg of RNA was transfected into the cells, 8 hours later RNA was isolated, 1 µg of RNA was used to make cDNA and assayed for gene expression by qRT-PCR). Due to cell number limitations, only a single time-point could be examined. Eight hours was chosen since the pDC-GEN2.2 gene expression peaked at this time-point. ELISAs and viral control were performed as described above for the pDC-GEN2.2 cell line.

### Statistics

Statistics were performed using Graphpad Prism statistical package. Student's T-test was used for comparisons amongst groups with more than 30 data points. Mann-Whitney non-parametric test was used for comparisons amongst groups with less than 30 data points. One sample t-tests or Mann-Whitney tests were used to compare fold increases of stimulated conditions with control conditions.

## Supporting Information

Figure S1 **IFNα2 has a relatively weak effect on IFN and PRR induction, compared to either TLR or PAMP stimulation.** A) IFNα2 (100 ng/mL) had a modest effect on most IFNs, failing to induce Type III IFNs. B) Of the PRR genes tested, only RIG-I was upregulated by IFNα. p values are the Wilcoxon signed rank result for the difference between the IFNα condition and media alone condition (dashed line) for each gene. Combined data for 5 independent experiments. * p<0.05 ** p<0.01 *** p<0.001 # p≤0.0001. Bars represent the mean and error bars are +/− SEM.
(TIF)

Figure S2 **Induction of interferon genes by transfection of the HCV PAMP or X-region control compared to the mock transfection.** A) Transfection of the pU/UC RNA into the pDC cell line induces robust IFN gene expression when compared to the mock transfected condition (dashed line). B) Transfection of the X-region RNA (Negative Control) into the pDC cell line induces low levels of IFN gene expression compared to the mock transfected condition (dashed line). Combined data from 5 independent experiments. Bars represent the mean and error bars are +/− SEM.
(TIF)

Figure S3 **RNaseL is not upregulated during the pDC-GEN2.2 response to the HCV PAMP.** A) RNaseL mRNA levels are not increased with pU/UC transfection nor are they increased over time. B) RNA gel of whole RNA from mock, X-region or pU/UC transfected pDC-GEN2.2 cells shows clear 28S and 18S rRNA bands suggesting that RNaseL is not activated by pU/UC transfection. C) Western blot of RNaseL in the pDC cell line shows no change of protein levels with HCV PAMP stimulation. D) Densitometry showed no differences amongst the conditions. Data are combined from 3 independent experiments. Gel and blot images are representative images of 3 independent experiments. Bars represent the mean and error bars are +/− SEM.
(TIF)

Figure S4 **HCV PAMP stimulated conditioned media upregulates IRF9 and STAT1 in Huh7.5.1 cells.** The top hits from the JAK/STAT PCR array were followed up by targeted qRT-PCR. As in **Table S1**, RNA was harvested and assayed 16 hours after addition of CM to infected Huh7.5.1 cells. p values are the Wilcoxon signed rank result for each gene compared to the X-region CM treatment from the same gene. * p<0.05 ** p<0.01 *** p<0.001 # p≤0.0001. Bars represent the mean and error bars are +/− SEM.
(TIF)

Figure S5 ***Ex vivo*** **conditioned media (CM) has IFN protein and controls viral replication.** ELISA data from the supernatants of HCV PAMP stimulated *ex vivo* pDCs for IFNα (A) and IL-29/IFNλ1 (B). C) Infected Huh7.5.1 cells were treated with *ex vivo* CM as described for pDC-GEN2.2 CM and HCV copy number was determined by qRT-PCR. Normalized HCV copy number is shown where the infection control condition HCV copy number is set to 1 and other conditions are expressed as normalized HCV copy number compared to infection control. Data is shown grouped by CC or non-CC genotype. Normalized HCV Copy Number = (Absolute copy number for condition/absolute copy number for infection control). p values are the Wilcoxon signed rank result for between the X-region and pU/UC CM conditions. Each graph for shows the total data from the 4 subjects assayed in **Figure 6**. * p<0.05 ** p<0.01 *** p<0.001 # p≤0.0001. Bars represent the mean and error bars are +/− SEM.
(TIF)

Figure S6 **Isolated** ***ex vivo*** **pDCs are 95+% pure and express classic pDC markers by flow cytometry.** A) Isolated *ex vivo* pDCs were HLA-DR+ BDCA-2+ CD123+ CD11c− BDCA-1−. B) Little contamination of CD56^+^ CD3^−^ (Natural Killer cells), CD19^+^ (B cells) and CD14^+^ (monocytes) in the pDC preparations. C) Isolated *ex vivo* pDCs express low levels of co-stimulation markers CD80 and CD86 but highly expressed CD44. D) *Ex vivo* pDCs express TLR9 but not TLR3.
(TIF)

Table S1 **HCV PAMP stimulated conditioned media upregulates the JAK/STAT pathway within hepatocytes.** HCV-infected Huh7.5.1 cells (24 hours of infection prior to CM addition) were assayed 16 hours after the addition of Conditioned Media from pU/UC or X-region stimulated pDC-GEN2.2 cells by PCR array for JAK/STAT genes expression changes. Shown are the genes that were differentially regulated in the cells treated with pU/UC CM by 2-fold or more compared to the X-region CM treated cells.
(DOC)
