# Correction: Hepatitis C Virus Pathogen Associated Molecular Pattern (PAMP) Triggers Production of Lambda-Interferons by Human Plasmacytoid Dendritic Cells

**DOI:** 10.1371/annotation/6382d059-925d-4af7-b6f2-3669fc68ddfa

**Published:** 2013-06-12

**Authors:** Amy E. L. Stone, Silvia Giugliano, Gretja Schnell, Linling Cheng, Katelyn F. Leahy, Lucy Golden-Mason, Michael Gale, Hugo R. Rosen

There are four changes that need to be made to this article. These changes affect the Introduction, References, Discussion and Figure Legends, respectively.

1. In the last sentence of the second paragraph of the Introduction, "a non-endocytic mechanism" should instead read "an exosomal mechanism".

2. Reference 13 is incorrect and should instead read:

"Dreux M, Garaigorta U, Boyd B, Decembre E, Chung J, et al. (2012) Short-range exosomal transfer of viral RNA from infected cells to plasmacytoid dendritic cells triggers innate immunity. Cell Host Microbe 12: 558-570."

3. In the second sentence of the fourth paragraph of the Discussion, reference 13 should instead refer to reference 12.

4. There is an error in the Figure 4 legend. The descriptions of panels A and B should be switched, so that the text instead reads as follows:

"A) RIG-I was knocked down in pDC-GEN2.2 cells using siRNA and then stimulated with the pU/UC or X-region RNA. When compared to the Scrambled siRNA condition, the RIG-I knock-down condition (mean knock-down 13% by PCR) produced significantly less IFN mRNA. Combined data from 3 independent experiments. Bars represent the mean and error bars are +/- SEM. p values are the Mann-Whitney result for the scrambled siRNA condition compared to the RIG-I siRNA condition. * p<0.05 ** p<0.01 *** p<0.001 # p=0.0001. B) Gene expression graphs show that, after normalization to the reference gene GAPDH and Mock transfected condition, when compared to untreated HCV RNA, the phosphatases-treated RNA induced less interferon gene production. Data are representative graph from 3 independent experiments. Bars represent the mean and error bars are +/- SD." 

