# Supplementary figures and images for: Hepatitis C Virus Pathogen Associated Molecular Pattern (PAMP) Triggers Production of Lambda-Interferons by Human Plasmacytoid Dendritic Cells
Source: PLoS Pathog. 2013 Apr 18;9(4):e1003316. doi: 10.1371/journal.ppat.1003316 (PMC3630164; doi:10.1371/journal.ppat.1003316)

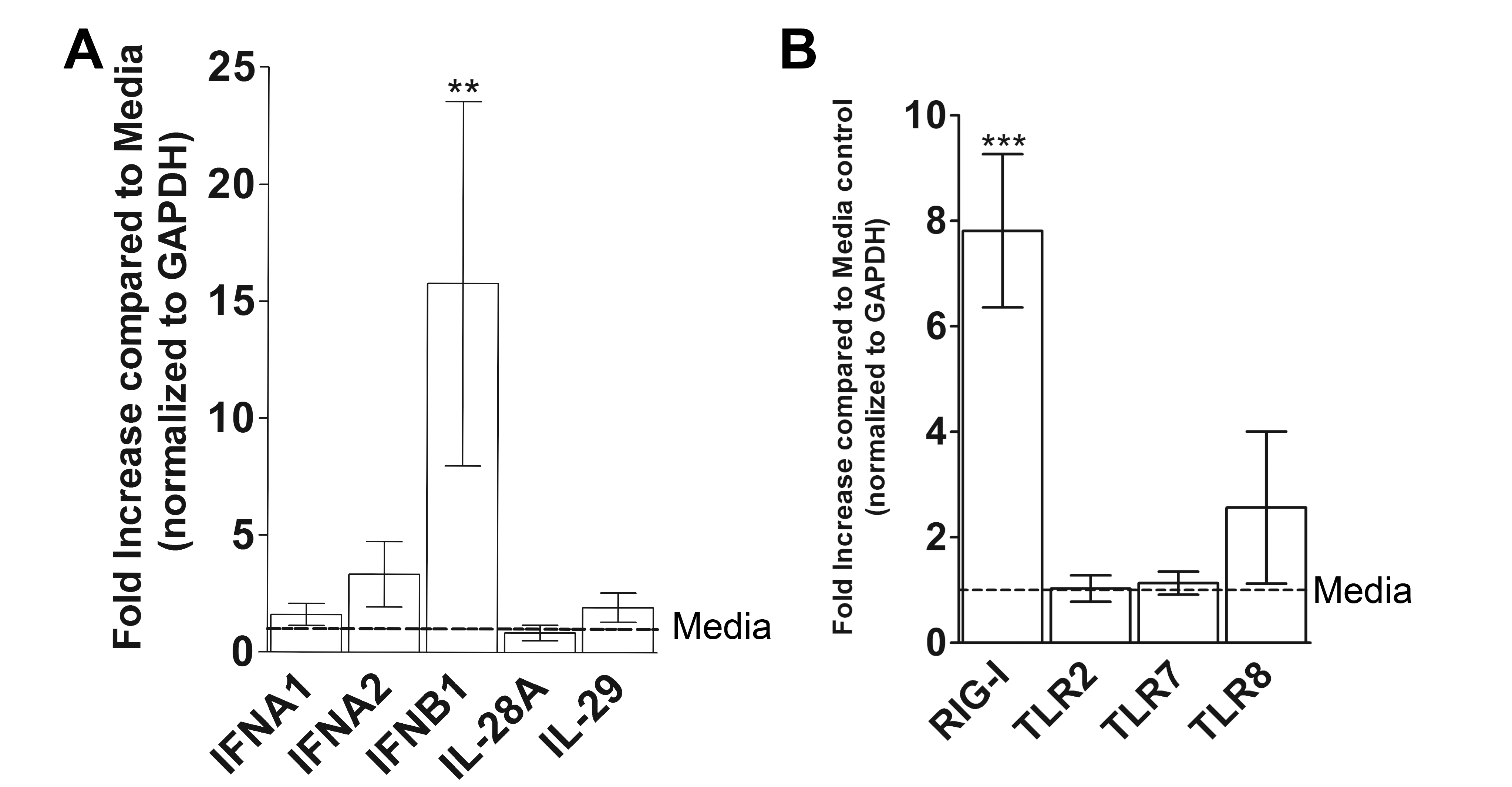

Supplement: Figure S1 — IFNα2 has a relatively weak effect on IFN and PRR induction, compared to either TLR or PAMP stimulation. A) IFNα2 (100 ng/mL) had a modest effect on most IFNs, failing to induce Type III IFNs. B) Of the PRR genes tested, only RIG-I was upregulated by IFNα. p values are the Wilcoxon signed rank result for the difference between the IFNα condition and media alone condition (dashed line) for each gene. Combined data for 5 independent experiments. * p<0.05 ** p<0.01 *** p<0.001 # p≤0.0001. Bars represent the mean and error bars are +/− SEM. (TIF) [file ppat.1003316.s001.tif]

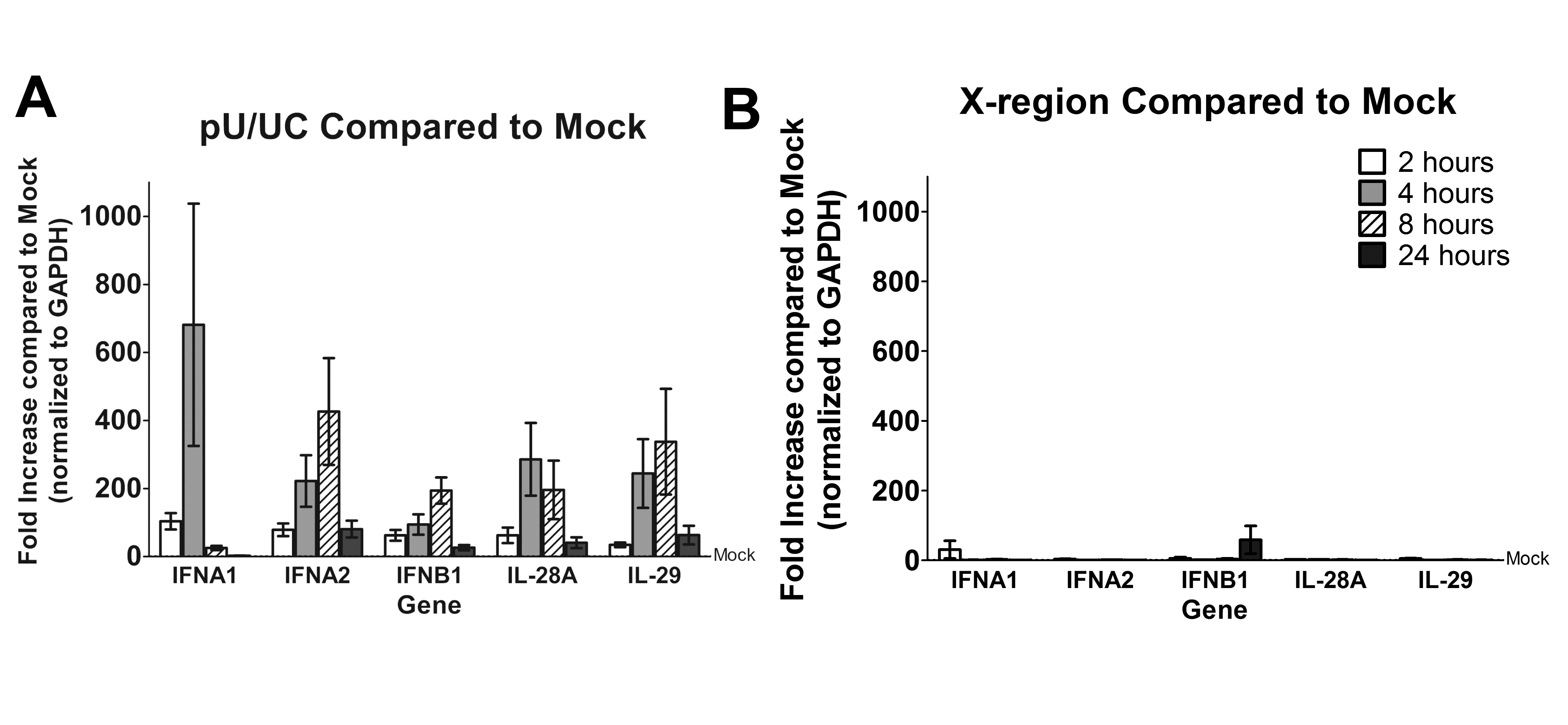

Supplement: Figure S2 — Induction of interferon genes by transfection of the HCV PAMP or X-region control compared to the mock transfection. A) Transfection of the pU/UC RNA into the pDC cell line induces robust IFN gene expression when compared to the mock transfected condition (dashed line). B) Transfection of the X-region RNA (Negative Control) into the pDC cell line induces low levels of IFN gene expression compared to the mock transfected condition (dashed line). Combined data from 5 independent experiments. Bars represent the mean and error bars are +/− SEM. (TIF) [file ppat.1003316.s002.tif]

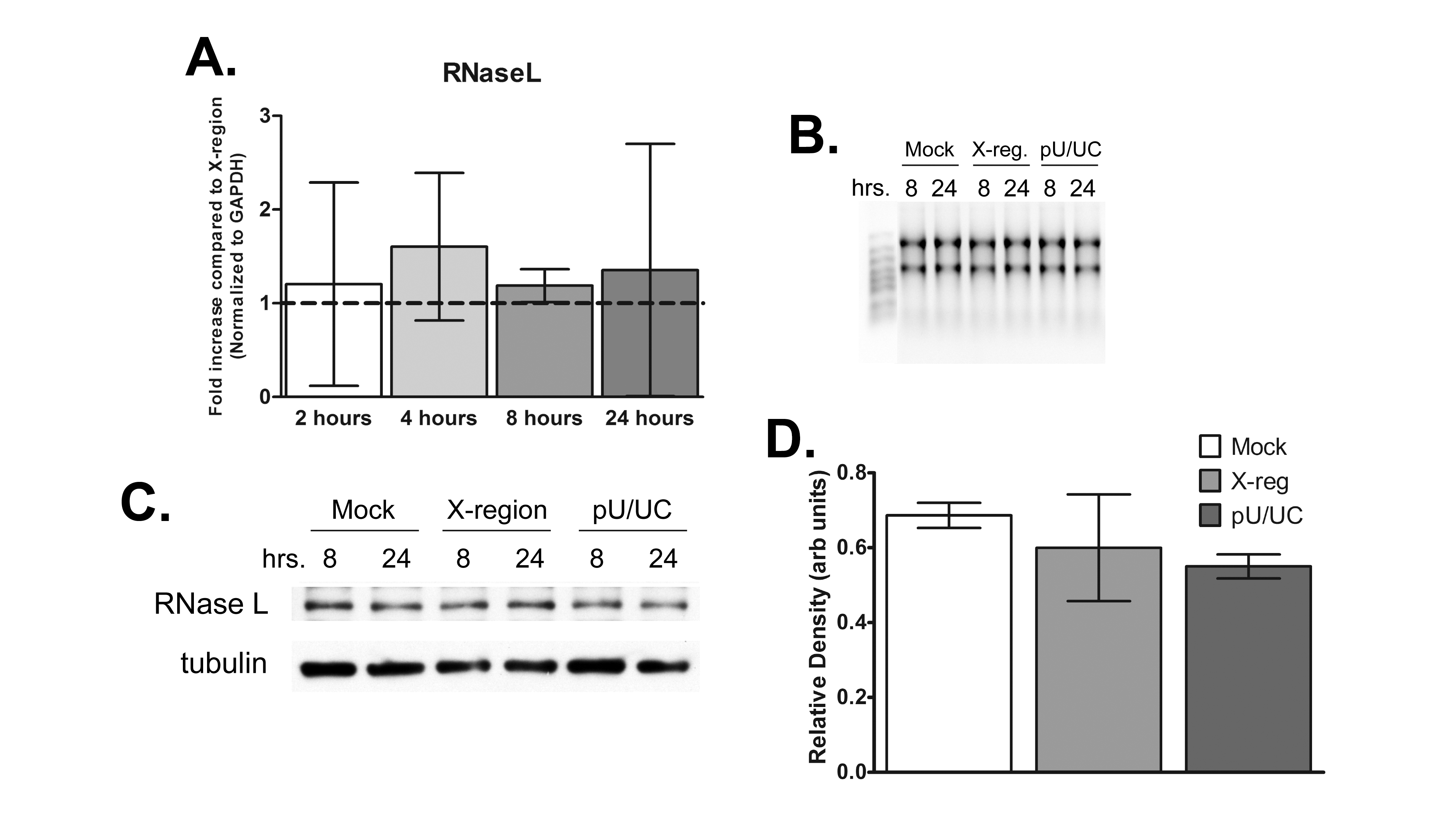

Supplement: Figure S3 — RNaseL is not upregulated during the pDC-GEN2.2 response to the HCV PAMP. A) RNaseL mRNA levels are not increased with pU/UC transfection nor are they increased over time. B) RNA gel of whole RNA from mock, X-region or pU/UC transfected pDC-GEN2.2 cells shows clear 28S and 18S rRNA bands suggesting that RNaseL is not activated by pU/UC transfection. C) Western blot of RNaseL in the pDC cell line shows no change of protein levels with HCV PAMP stimulation. D) Densitometry showed no differences amongst the conditions. Data are combined from 3 independent experiments. Gel and blot images are representative images of 3 independent experiments. Bars represent the mean and error bars are +/− SEM. (TIF) [file ppat.1003316.s003.tif]

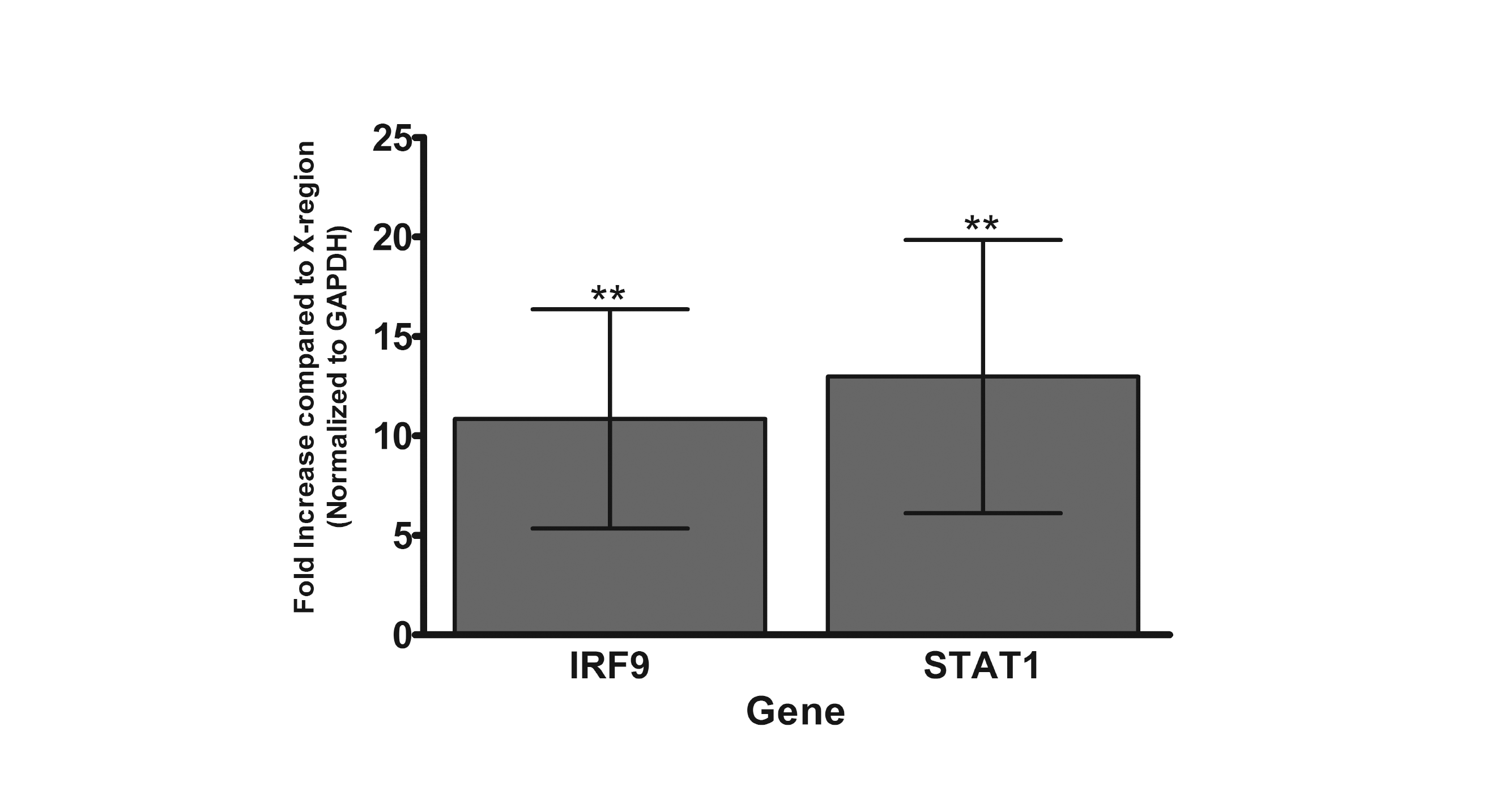

Supplement: Figure S4 — HCV PAMP stimulated conditioned media upregulates IRF9 and STAT1 in Huh7.5.1 cells. The top hits from the JAK/STAT PCR array were followed up by targeted qRT-PCR. As in Table S1, RNA was harvested and assayed 16 hours after addition of CM to infected Huh7.5.1 cells. p values are the Wilcoxon signed rank result for each gene compared to the X-region CM treatment from the same gene. * p<0.05 ** p<0.01 *** p<0.001 # p≤0.0001. Bars represent the mean and error bars are +/− SEM. (TIF) [file ppat.1003316.s004.tif]

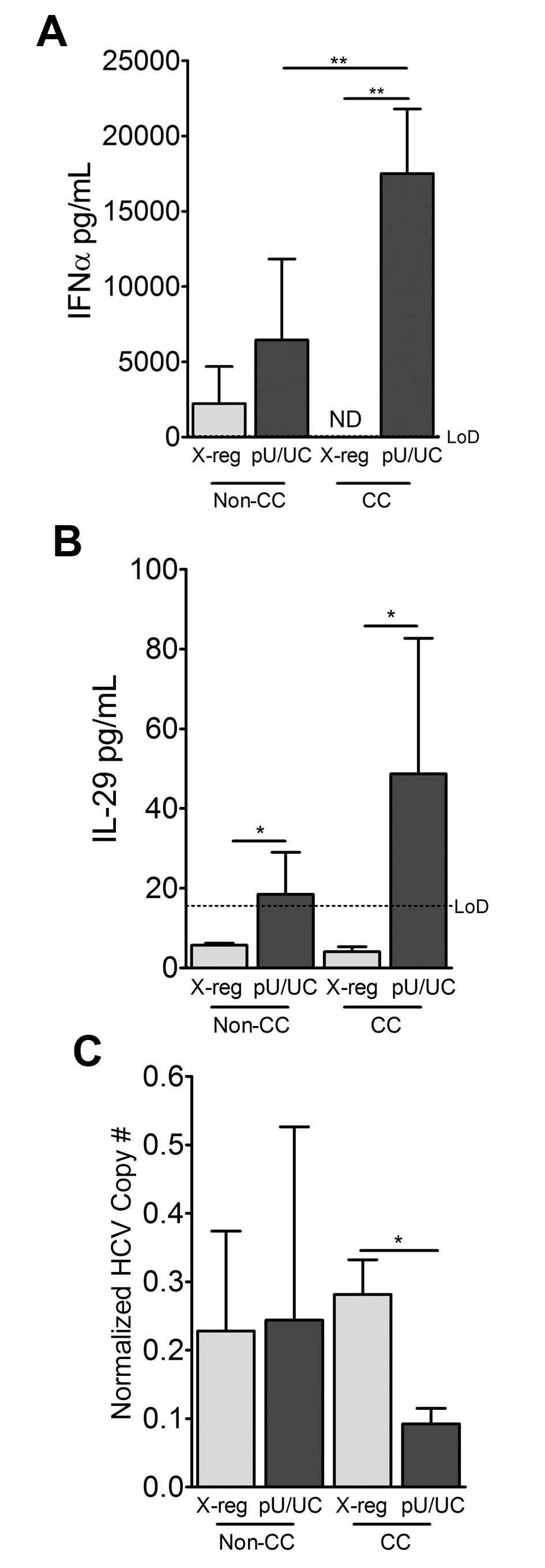

Supplement: Figure S5 — Ex vivo conditioned media (CM) has IFN protein and controls viral replication. ELISA data from the supernatants of HCV PAMP stimulated ex vivo pDCs for IFNα (A) and IL-29/IFNλ1 (B). C) Infected Huh7.5.1 cells were treated with ex vivo CM as described for pDC-GEN2.2 CM and HCV copy number was determined by qRT-PCR. Normalized HCV copy number is shown where the infection control condition HCV copy number is set to 1 and other conditions are expressed as normalized HCV copy number compared to infection control. Data is shown grouped by CC or non-CC genotype. Normalized HCV Copy Number = (Absolute copy number for condition/absolute copy number for infection control). p values are the Wilcoxon signed rank result for between the X-region and pU/UC CM conditions. Each graph for shows the total data from the 4 subjects assayed in Figure 6 . * p<0.05 ** p<0.01 *** p<0.001 # p≤0.0001. Bars represent the mean and error bars are +/− SEM. (TIF) [file ppat.1003316.s005.tif]

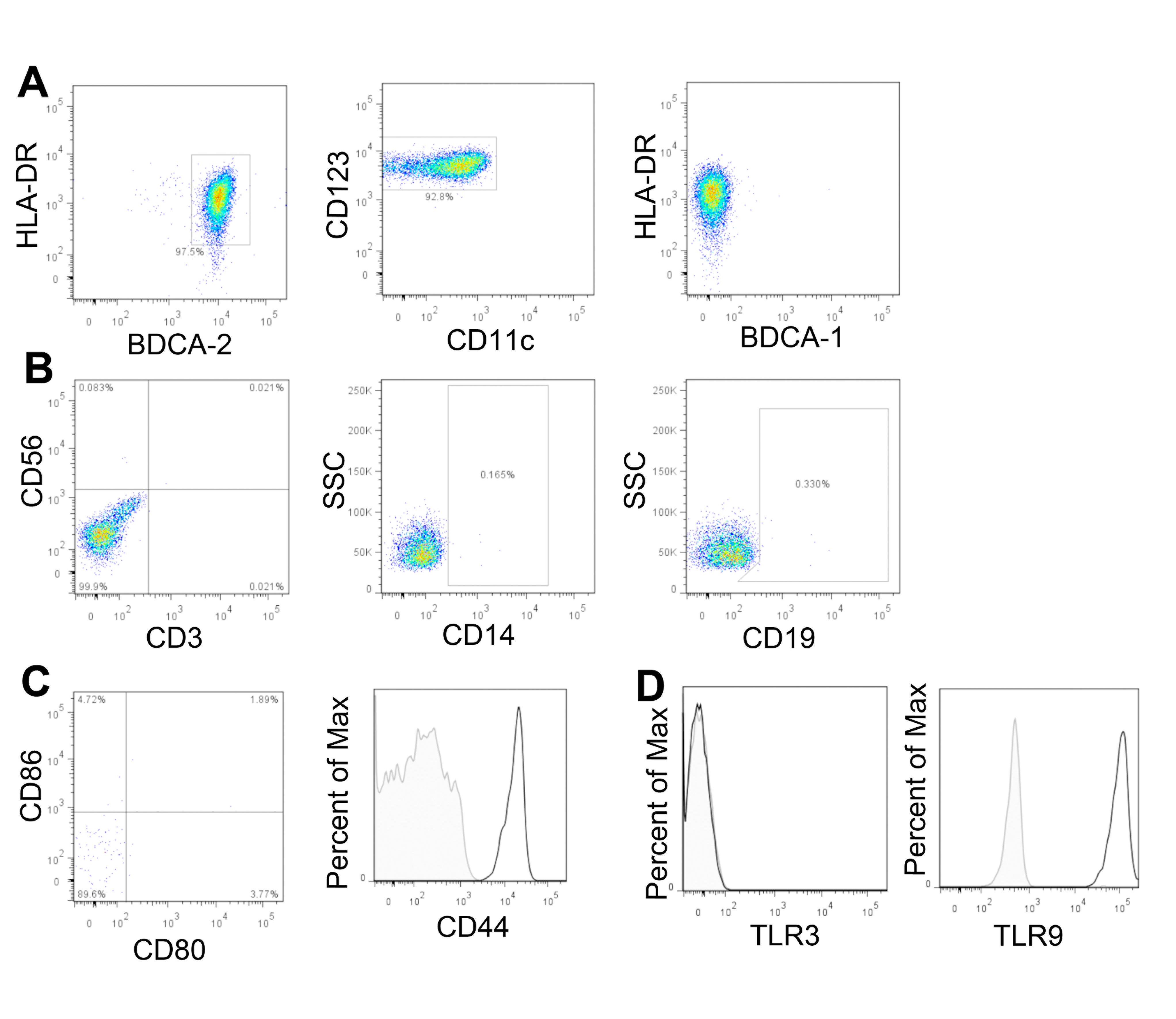

Supplement: Figure S6 — Isolated ex vivo pDCs are 95+% pure and express classic pDC markers by flow cytometry. A) Isolated ex vivo pDCs were HLA-DR+ BDCA-2+ CD123+ CD11c− BDCA-1−. B) Little contamination of CD56+ CD3− (Natural Killer cells), CD19+ (B cells) and CD14+ (monocytes) in the pDC preparations. C) Isolated ex vivo pDCs express low levels of co-stimulation markers CD80 and CD86 but highly expressed CD44. D) Ex vivo pDCs express TLR9 but not TLR3. (TIF) [file ppat.1003316.s006.tif]
