# Supplementary material for: Hepatitis C Virus Pathogen Associated Molecular Pattern (PAMP) Triggers Production of Lambda-Interferons by Human Plasmacytoid Dendritic Cells
Source: PLoS Pathog. 2013 Apr 18;9(4):e1003316. doi: 10.1371/journal.ppat.1003316 (PMC3630164; doi:10.1371/journal.ppat.1003316)
Supplement: Table S1 — HCV PAMP stimulated conditioned media upregulates the JAK/STAT pathway within hepatocytes. HCV-infected Huh7.5.1 cells (24 hours of infection prior to CM addition) were assayed 16 hours after the addition of Conditioned Media from pU/UC or X-region stimulated pDC-GEN2.2 cells by PCR array for JAK/STAT genes expression changes. Shown are the genes that were differentially regulated in the cells treated with pU/UC CM by 2-fold or more compared to the X-region CM treated cells. (DOC) [file ppat.1003316.s007.doc]

**Supplemental table 1:** HCV PAMP stimulated conditioned media upregulates the JAK/STAT pathway

| Gene Name | Fold change |
| --- | --- |
| PIK3CD | 0.50 |
| EPOR | 0.50 |
| ERBB2 | 0.50 |
| 18S | 0.50 |
| STAT2 | 2.00 |
| SUMO1 | 2.01 |
| STAT3 | 2.01 |
| GUSB | 2.01 |
| MAP2K4 | 2.01 |
| JUN | 2.01 |
| AKT3 | 2.01 |
| PIK3CB | 2.01 |
| HRAS | 2.01 |
| PSMA3 | 2.01 |
| PIK3R1 | 2.02 |
| PIK3CA | 2.02 |
| SP1 | 2.02 |
| EP300 | 2.02 |
| MYC | 2.02 |
| SOS1 | 2.02 |
| FGF2 | 2.02 |
| PTPN6 | 2.02 |
| IFNAR1 | 2.02 |
| ELP2 | 2.03 |
| STAT5B | 2.03 |
| THPO | 2.03 |
| PTPN11 | 2.03 |
| JAK1 | 2.03 |
| UBC | 2.03 |
| RAF1 | 2.04 |
| STAM | 2.04 |
| MAP2K3 | 2.05 |
| PRLR | 2.05 |
| JAK2 | 2.11 |
| IFNA2 | 4.92 |
| IFNA1 | 6.31 |
| IRF9 | 7.99 |
| STAT1 | 8.04 |
